# Endoscopic Ultrasound‐Guided Bile Duct Drainage Enhances Oncological Systemic Therapy Initiation and Capability in Patients With Non‐Resectable Malignant Distal Bile Duct Obstructions Compared to Percutaneous Transhepatic Biliary Drainage

**DOI:** 10.1002/deo2.70262

**Published:** 2025-12-08

**Authors:** Thomas Roland Heiduk, Andre Sasse, Volker Ellenrieder, Lukas Hiebel, Richard Friedemann Knoop, Golo Petzold, Ahmad Amanzada

**Affiliations:** ^1^ Clinic of Gastroenterology, Gastrointestinal Oncology and Endocrinology University Medical Center Göttingen Göttingen Germany

**Keywords:** bile duct drainage, bile duct obstruction, neoplasia, time to treatment, ultrasound‐guided

## Abstract

**Introduction:**

Endoscopic retrograde cholangiopancreaticography (ERCP) is the gold standard for bile duct drainage in non‐resectable malignant distal bile duct obstruction with jaundice in pancreaticobiliary and duodenal neoplasia. Failed ERCP is either followed by endoscopic ultrasound‐guided biliary drainage (EUS‐BD) or percutaneous transhepatic biliary drainage (PTBD) as an alternative procedure. This study compares the ability to initiate and continue oncological systemic therapy in patients treated with EUS‐BD versus PTBD.

**Methods:**

In this retrospective, comparative cohort study, 96 consecutive patients were analyzed. Oncological, demographic, and laboratory parameters were examined, focusing on the capability to initiate chemotherapy and the time interval from intervention to chemotherapy initiation.

**Results:**

The EUS‐BD group showed a greater reduction in serum bilirubin levels before chemotherapy (2.2 mg/dL vs. 3.9 mg/dL, *p* = 0.04) and had a significantly greater degree of change in bilirubin levels after 10 days (70% vs. 30%, *p* = 0.01). More patients in the EUS‐BD cohort received chemotherapy (69% vs. 48%, *p* = 0.04), and the interval between intervention and chemotherapy initiation was shorter (10 vs. 17 days, *p* = 0.02). The type and number of chemotherapies administered did not differ significantly between the groups (*p* = 0.43; *p* = 0.50; *p* = 0.12). Reinterventions and complication rates were significantly lower in the EUS‐BD cohort (6% vs. 60%, *p* = 0.004 and 56% vs. 92%, *p* = 0.004).

**Conclusion:**

EUS‐BD appears superior to PTBD in facilitating the initiation of oncological systemic therapy, with fewer complications and a shorter time to treatment initiation. Further multicenter studies are needed to confirm these findings.

## 1 Introduction

Malignant diseases of the distal bile duct and the pancreatic head frequently cause obstructive jaundice. Endoscopic retrograde cholangiopancreatography (ERCP), with its possibilities of therapeutic interventions, remains the gold standard to access the bile duct in these patients. Unfortunately, biliary access and cannulation can fail in up to 5%–15% of procedures even in high‐volume expert centers [1, 2, 3, 4].

Difficult anatomical features, such as in patients post‐pancreaticoduodenectomy or following pylorus‐preserving pancreaticoduodenectomy, can further complicate ERCP procedures [5]. In such cases, percutaneous transhepatic biliary drainage (PTBD) was traditionally used to provide an alternative means of biliary drainage, but PTBD is associated with several complications, including infections, catheter‐related issues, and a poor quality of life for patients [6, 7].

If ERCP and PTBD were unsuccessful, a surgical biliodigestive anastomosis for bile drainage had to be performed as a last alternative. However, this is associated with high morbidity and mortality [8, 9, 10].

In contrast, endoscopic ultrasound (EUS)‐guided biliary drainage (EUS‐BD) has emerged as a promising alternative. Using EUS guidance, usually a covered metal stent is placed from the stomach or duodenum into the bile duct, creating an internal drainage pathway. This minimally invasive procedure has been shown to offer several advantages over PTBD, including better quality of life, fewer complications, and reduced long‐term costs [11, 12, 13, 14, 15, 16, 17, 18].

However, while several studies have compared the technical and clinical outcomes of EUS‐BD and PTBD, there is limited data on the impact of these procedures on the initiation and continuation of systemic oncological therapy, which is a crucial aspect of treatment for these patients. This study aims to compare the oncological therapy capability, the time interval between intervention and chemotherapy initiation, and the incidence of adverse events in patients treated with either EUS‐BD or PTBD.

## 2 Methods

### 2.1 Study Design and Patient Selection

This is a retrospective, single‐center, comparative cohort study. Data were sourced from internal hospital documentation systems and analyzed from 2010 to 2023. All patients who underwent EUS‐BD or PTBD in this time period were analyzed, and the cohort was formed by consecutive patients.

Inclusion criteria were patients with all of the following below: Underlying non‐resectable malignant disease; jaundice due to distal bile duct obstruction; failed ERCP; patients with EUS‐BD bile duct drainage; patients with PTBD; patients with available laboratory parameters (blood count, bilirubin, and CRP) before and after the procedures and patients with available data about the date of first chemotherapy application after intervention as well as if chemotherapy administration was possible.

Exclusion criteria were patients who could not be clearly assigned to any group, patients with benign biliary obstruction, and patients with missing laboratory parameters or date of chemotherapy administration.

After the criteria had been followed, 48 patients in the EUS‐BD cohort and 48 patients in the PTBD cohort remained. Matching was not performed.

The treatment of the included patients was completed prior to the study. Due to its retrospective design, patients were not re‐contacted. Consequently, only existing data and medical records were analyzed.

Blood samples were taken routinely during the patient's hospital stay. The included parameters were assessed up to 3 days before the procedure and were monitored until the moment of chemotherapy administration. The latest parameters are not older than 3 days until the moment of oncologic systemic therapy.

### 2.2 Definitions

Technical success was defined as the correct transmural or transpapillary stent placement resulting in bile flow into the gastrointestinal tract based on the current ESGE Guidelines. Clinical success was defined as a post‐procedural reduction in bilirubin levels of 50%–75% from baseline from hospital administration until the moment of chemotherapy administration. Both definitions are based on the current ESGE Guideline [19], with the latter slightly adjusted to our study time interval.

Adverse events were defined as any undesirable events related to the procedure, as there is no universal definition in the ESGE Guidelines. Common reported adverse events as hemorrhage (characterized by a hemoglobin drop of more than 2 g/dL within 24 h), abdominal pain, infection, dislocation, and other events such as extravasation, nausea, and vomiting, were included.

### 2.3 Medical Devices

The endosonographic guided biliary drainage procedure and the PTBD were carried out by expert endoscopists in our clinic with years of experience in gastrointestinal endoscopy.

The endoscopes used in the EUS‐BD were from our hospital's supplier, OLYMPUS, model GF‐UCT180. All endoscopes were regular endosonographic endoscopes without any adjustments. The ultrasound processor used was EU.ME2 Premier Plus from our supplier, OLYMPUS, as well. For endosonographic tract creation, we used a 19‐A COOK MEDICAL Echotip Ultra HD access needle, a VisiGlide 2 single‐use guidewire by OLYMPUS (4500 mm in length and 0.025 inch in diameter), as well as the Will Ring knife by MTW for cystotomy. A dilator was not used by the endoscopists.

The stents used were up to the endoscopist's choice. Metal‐ as well as plastic‐stents were used in this study to achieve biliary flow.

PTBD was performed using a combined ultrasonographic and fluoroscopic approach. All PTBDs included in this study were performed by creating an internal‐external drainage by either an 8‐ or 10‐Fr (PerkuBil ´´Münchner Drainage``) plastic stent.

### 2.4 Overall Strategy of Endoscopic Approach

The overall strategy for biliary drainage was not determined by the primary tumor site. A substantial shift in institutional expertise regarding endosonographic interventions occurred during the study period. Until late 2019, PTBD represented the standard approach to biliary drainage in our department. Thereafter, EUS‐BD became the preferred modality, and patients routinely underwent endosonographic intervention prior to consideration of PTBD.

### 2.5 Outcome Measures

The primary outcomes were the ability to receive oncological systemic therapy and the time interval from intervention to initiation of chemotherapy. Secondary outcomes included total serum bilirubin levels before and after the procedure, as well as reintervention and complication rates.

### 2.6 Statistical Analysis

Demographic, oncological, and laboratory parameters, as well as adverse events related to the interventions, were analyzed for all patients.

Descriptive statistics were used to summarize baseline characteristics. The categorical data of both groups were compared using the Chi‐square test or Fisher's exact test, and continuous variables were analyzed using the t‐test or Mann‐Whitney U‐test. A p‐value of <0.05 was considered statistically significant. To control the expected proportion of false discoveries, we applied the Benjamini‐Hochberg procedure to further adjust our *p*‐values. All analyses were performed using SPSS Version 30.

## 3 Results

### 3.1 Baseline Characteristics

A total of 96 patients were included, with 48 in the EUS‐BD group and 48 in the PTBD group. The two cohorts were comparable in terms of gender, Eastern Cooperative Oncology Group (ECOG) status, age, and serum bilirubin levels before the intervention. A statistically significant difference was found in the underlying malignancies between the two groups (*p* = 0.01). The EUS‐BD group had a significantly higher proportion of pancreatic carcinomas (*p* = 0.007), while the PTBD group had more cases of metastatic obstruction (*p* = 0.02). There was no significant difference in the distribution of bile duct carcinoma between the two groups (*p* = 0.18). Cholangitis was more present in the PTBD group (42% vs. 17%, *p* = 0.01). Both cohorts expressed a slight difference in the type of obstruction (*p* = 0.05); while the EUS‐BD cohort had more distal obstructions (30% vs. 20%), the PTBD cohort consisted of more hilar obstructions (15% vs. 27%). Both groups expressed a significant difference in the bile duct diameters, which were punctured to achieve biliary flow (*p* = 0.02); the EUS‐BD group showed a greater CBD diameter (18 mm vs. 12 mm; *p* = 0.02) while the PTBD cohort presented a wider PBD diameter (5 mm vs. 7 mm; *p* = 0.02) when comparing each group to each other.

There were 26 (54%) EUS‐CD patients and 22 (46%) EUS‐HG patients in the EUS‐BD cohort. The right liver lobe was punctured 22 (54%) times, while the left liver lobe was punctured 22 (46%) times in the PTBD cohort. 42 (88%) metal stents and 6 (12%) plastic stents were used in the EUS‐BD cohort to achieve biliary drainage, as shown in Table 1.

**TABLE 1:** Basic characteristics.

	EUS‐BD *n* = 48	PTBD *n* = 48	Total *n* = 96	p value (EUS‐BD vs. PTBD)
Women, *n* (%)	23 (48)	17(35)	40(42)	0.30
Age, median (min‐max), years	68 (44–86)	66 (39–90)	67 (39–90)	0.90
ECOG, median (min‐max)	1 (0–3)	0.5 (0–3)	1 (0–3)	0.90
**Underlying disease**				
Pancreatic carcinoma, *n* (%)	33 (69)	18 (37)	51 (53)	**0.007** ^*^
Bile duct carcinoma, *n* (%)	6 (12)	11 (23)	17 (18)	0.180
Metastases, *n* (%)	9 (19)	19 (39)	27 (29)	**0.020** ^*^
Cholangitis, *n* (%)	8 (17)	20 (42)	28 (29)	**0.01** ^*^
**Type of obstruction**				
Distal, *n* (%)	29 (30)	19 (20)	48 (50)	0.05
Hilar, *n* (%)	14 (15)	26 (27)	40 (42)	
Middle, *n* (%)	5 (5)	3 (3)	8 (8)	
**Bile ducts' diameter at the time of puncture**				
CBD mm, median (min–max)	18 (4–23)	12 (8–18)		**0.02** ^*^
PBD mm, median (min–max)	5 (4–12)	7(4–10)		**0.02** ^*^
**Bile duct puncture sites, types of stents used, and stent size**				
*Types of stents used*				
*Metal, n (%)*	42 (88)	N/A		
fcSEMS, *n* (%)	17 (35)	N/A		
6 cm/10 mm, *n* (%)	7 (15)	N/A		
6 cm/ 8 mm, *n* (%)	1 (2)	N/A		
LAMS, *n* (%)				
Hot‐Axios 8/8 mm, *n* (%)	7 (15)	N/A		
Hot‐Axios 10/10 mm, *n* (%)	2 (2)	N/A		
scSEMS, n (%)	25 (53)	N/A		
6 cm/10 mm	17 (35)	N/A		
8 cm/10 mm	7 (15)	N/A		
8 cm/ 8 mm	1 (2)	N/A		
*Plastic, n (%)*	6 (12)	N/A		
8 Fr/ 7 cm, *n* (%)	4 (8)	N/A		
8 Fr/ 9 cm, *n* (%)	2 (4)	N/A		
**Puncture site**				
*EUS‐CD, n (%)*	26 (54)	N/A		
*EUS‐HG, n (%)*	22 (46)	N/A		
Right liver lobe, *n* (%)	N/A	26 (54)		
Left liver lobe, *n* (%)	N/A	22 (46)		

Abbreviations: CBD, common bile duct; ECOG, Eastern Cooperative Oncology Group; EUS, endoscopic ultrasound; EUS‐BD, endoscopic ultrasound guided bile duct drainage; EUS‐CD, endoscopic ultrasound guided choledochoduodenostomy; EUS‐HG, endoscopic guided hepaticogastrostomy; Fr, French; LAMS, lumen‐apposing metal stent; PBD, peripheral bile duct; PTBD, percutaneous transhepatic bile drainage.

* adjusted *p*‐value.

### 3.2 Overall Outcomes

The technical success rate was slightly higher in the EUS‐BD cohort than in the PTBD cohort (100% vs. 90%; *p* = 0.05). Furthermore, the procedure time at the EUS‐BD cohort was significantly shorter compared to the PTBD cohort (48 min vs. 67 min, *p* = 0.01). Additionally, the EUS‐BD group had significantly fewer reinterventions and complications compared to the PTBD group (6% vs. 60%, *p* = 0.004 for reinterventions; 56% vs. 92%, *p* = 0.005 for complications). Infections were notably lower in the EUS group than in the PTBD group (22% vs. 29%, *p* = 0.02), as shown in Table 2.

**TABLE 2:** Procedure comparisons, capability for oncological systemic therapy, and reintervention rates following biliary drainage.

	EUS‐BD *n* = 48	PTBD *n* = 48	Total *n* = 96	*p*‐Value (EUS‐BD vs. PTBD)
*Procedure comparisons*				
Procedure time, median (min–max) minutes	48 (29–88)	67 (49–185)		**0.01** ^*^
Technical success, *n* (%)	48 (100)	43 (90)		0.05
Adverse events, *n* (%)	27 (56)	44 (92)	71 (74)	**0.005** ^*^
Type and number of complications that occurred in the 71 patients				

	EUS‐BD *n* = 37	PTBD *n* = 65	Total *n* = 102	
Haemorrhage, *n* (%)	4 (11)	6 (9)	10 (10)	0.74
Abdominal pain, *n* (%)	10 (27)	19 (29)	29 (29)	0.07
Infections, *n* (%)	8 (22)	19 (29)	27 (27)	**0.02** ^*^
Dislocation, *n* (%)	8 (22)	6 (9)	14 (14)	0.77
Other complications, *n* (%)	7 (18)	15 (24)	22 (22)	0.09
*Chemotherapy specificities*				
Chemotherapy capability after intervention, *n* (%)	33 (69)	23 (48)	56 (58)	**0.04** ^*^
First line regimen, *n* (%)	33 (100)	23 (100)	56 (100)	—
Interval between intervention and chemotherapy administration, median (min–max) in days	10 (3–58)	17 (6–91)	15 (3–91)	**0.02** ^*^
Number of patients with at least one re‐intervention, *n* (%)	3 (6)	29 (60)	32 (33)	**0.004** ^*^

Abbreviations: EUS, endoscopic ultrasound; EUS‐BD, endoscopic ultrasound‐guided bile duct drainage; PTBD, percutaneous transhepatic bile drainage.

* adjusted *p*‐value.

### 3.3 Oncological Therapy Capability and Chemotherapy Initiation

**FIGURE 1:**
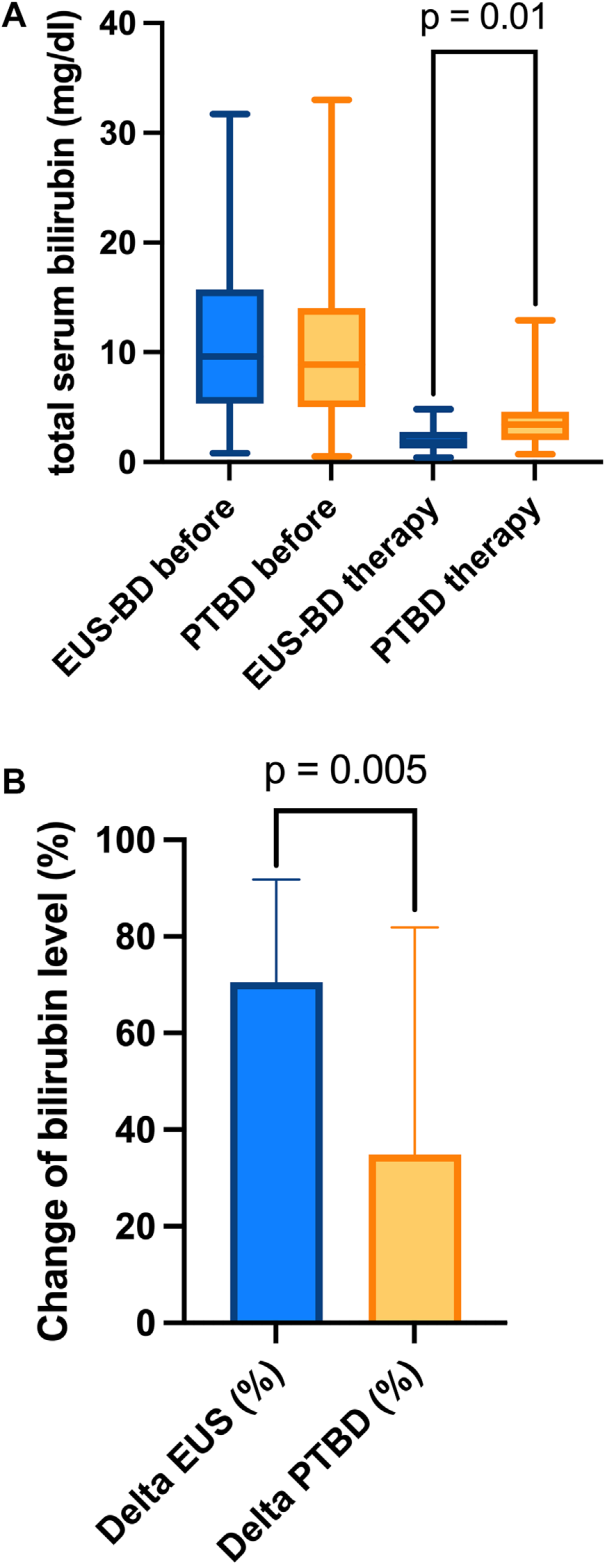
(a) Total serum bilirubin levels before and on the day of chemotherapy application in mg/dL, including adjusted *p*‐value. (b) Change of bilirubin levels in % after 10 days after the first intervention, including adjusted *p*‐value.

The EUS‐BD group showed a significantly greater reduction in serum bilirubin levels before chemotherapy initiation compared to the PTBD group (2.2 mg/dL vs. 3.9 mg/dL, *p* = 0.01), as shown in Figure 1a. Additionally, the degree of change in bilirubin levels was significantly higher after 10 days in the EUS‐BD group than in the PTBD group (70% vs. 30%, *p* = 0.01), as visualized in Figure 1b. Furthermore, a significantly higher proportion of patients in the EUS‐BD group were able to receive oncological systemic therapy (69% vs. 48%, *p* = 0.04). The mean interval between intervention and chemotherapy initiation was also significantly shorter in the EUS‐BD group (10 days vs. 17 days, *p* = 0.02), as shown in Table 2.

Both cohorts differed significantly in terms of underlying malignancy (*p* = 0.04). But all patients undergoing chemotherapy received a first‐line regimen in both cohorts. No significant differences were observed in the type or number of chemotherapies administered among the subgroups of pancreatic carcinoma, bile duct carcinoma, and metastasis (*p* = 0.43, *p* = 0.50, *p* = 0.12) as shown in Table 3.

**TABLE 3:** Chemotherapy eligibility in relation to underlying malignant disease for each group.

	EUS‐BD = 33	PTBD = 23	*p*‐Value
Pancreatic carcinoma, *n* (%)	25 (75)	10 (55)	**0.04** ^*^
Bile duct carcinoma, *n* (%)	2 (33)	5 (45)	
Metastases, *n* (%)	6 (66)	8 (42)	
*Chemotherapy initiation in pancreatic adenocarcinoma patients after intervention*			
Number and type of chemotherapy administration	EUS‐BD = 25	PTBD = 10	*p*‐Value
FOLFIRINOX, *n* (%)	18 (72)	5 (50)	
Gemcitabine+Paclitaxel/other, *n* (%)	7 (28)	5 (50)	0.43
*Chemotherapy initiation in bile duct carcinoma patients after intervention*			
Number and type of chemotherapy administration	EUS‐BD = 2	PTBD = 5	*p*‐Value
Gemcitabine/Cisplatin, *n* (%)	2 (100)	4 (80)	
Other, *n* (%)	0 (0)	1 (20)	0.50
*Chemotherapy initiation in patients with metastases after intervention*			
Number and type of chemotherapy administration	EUS‐BD = 6	PTBD = 8	*p*‐Value
Carboplatin/Etoposide/FLOT, *n* (%)	2 (33)	2 (25)	
Other, *n* (%)	4 (66)	6 (75)	0.12

Abbreviations: EUS, endoscopic ultrasound; EUS‐BD, endosonographic bile duct drainage; PTBD, percutaneous transhepatic bile drainage.

* adjusted *p*‐value.

## 4 Discussion

In patients with malignant obstructive jaundice, ERCP remains the standard of care for accessing the biliary tree, with success rates exceeding 90% [20]. However, ERCP failure can occur in 5%–15% of cases [21, 22]. In these situations, alternative biliary drainage procedures such as PTBD or EUS‐BD are used.

A major concern with long‐term PTBD is the risk of complications such as drain blockage, dislocation, cholangitis, and the need for repeated interventions, leading to extended hospital stays [23]. Additionally, PTBD often necessitates the use of an external drain, which significantly diminishes the patient's quality of life [24, 25]. On the other hand, EUS‐BD has emerged as a safe, minimally invasive alternative that leaves the patient with an internal bile duct stent, improving both the patient's quality of life and clinical outcomes [25, 26].

To the best of our knowledge, this is the first study to specifically evaluate oncological systemic therapy eligibility and the time interval to chemotherapy initiation following bile duct drainage in these patient populations. The key findings from this study include: (1) the EUS‐BD group showed a significantly greater reduction in serum bilirubin levels before chemotherapy initiation compared to the PTBD group, (2) the EUS‐BD cohort experienced a significantly shorter time interval between intervention and chemotherapy initiation, (3) a higher chemotherapy eligibility in the EUS‐BD cohort, (4) no significant differences were observed between the two groups regarding the type or number of chemotherapy regimens administered, suggesting that the improved chemotherapy initiation in the EUS‐BD group was not due to differences in the chemotherapy protocols used and (5) a lower reintervention and complication rates in the EUS‐BD cohort.

Our data show that the EUS‐BD group experienced a more significant reduction in total serum bilirubin levels before chemotherapy initiation compared to the PTBD group. For instance, a systematic meta‐analysis by Hassan and Gadour [25] examined serum bilirubin level reduction within 7 days post‐drainage, finding similar improvements in both EUS‐BD and PTBD groups.

One of the key findings mentioned above was the higher chemotherapy eligibility rate in the EUS‐BD cohort compared to the PTBD cohort. Several factors could explain this observation. First, EUS‐BD is a less invasive procedure compared to PTBD, which might result in a quicker recovery time, enabling earlier initiation of chemotherapy. Chemotherapy eligibility is often contingent on the patient's overall condition and ability to tolerate treatment, and less invasive procedures may reduce procedural risks and post‐operative complications, allowing for a more timely chemotherapy regimen.

While one might speculate that differences in chemotherapy regimens could introduce bias, we further analyzed the data according to the underlying malignancy and the type and number of chemotherapy regimens administered. There were no significant differences in the type or number of chemotherapies initiated among the pancreatic carcinoma, bile duct carcinoma, and metastasis groups. Furthermore, all patients receiving chemotherapy underwent a first‐line regimen in both groups. This suggests that the EUS‐BD cohort not only received oncological systemic therapy more frequently and more quickly, but also had similar chemotherapy regimens administered compared to the PTBD cohort.

The significantly lower reintervention and complication rates in the EUS‐BD cohort align with findings from several previous studies. A systematic review by Hassan and Gadour [25] also demonstrated a lower incidence of complications and reinterventions with EUS‐BD compared to PTBD. This is likely due to the already mentioned less invasive nature of EUS‐BD, as well as the avoidance of long‐term external drainage, which can lead to complications such as infections, dislocation, and cholangitis [23, 24, 25].

Interestingly, our data show a slightly higher technical success rate in the EUS‐BD cohort, which contrasts with some previous reports that found comparable technical success rates for both procedures [27, 28]. This difference may reflect the expertise of the operators, as well as the centers' experience in performing these procedures. Binda et al. [29] have demonstrated that the technical and clinical success rates of procedures like EUS‐BD are significantly influenced by the volume of cases handled by a center and the experience of the practitioners.

The study's retrospective, single‐center design represents a significant limitation. Due to this, long‐term outcomes like quality of life and overall survival were not assessed within the present analysis. Additionally, the relatively small cohort sizes (48 patients in each group) and the heterogeneity of the underlying malignancies (pancreatic cancer, bile duct cancer, metastasis) may limit the generalizability of the results. The distribution of malignant diseases between the two groups differed significantly, which could introduce bias.

Notably, our *p*‐values were adjusted using the Benjamini‐Hochberg procedure. All our significant results stayed significant even after multiple testing was performed.

In conclusion, the results of this cohort study suggest that EUS‐BD may be superior to PTBD in patients with non‐resectable, malignant distal bile duct obstruction, particularly in terms of oncological therapy eligibility and the interval between intervention and subsequent systemic oncological therapy. Moreover, patients in the EUS‐BD group experienced fewer adverse effects and required fewer reinterventions compared to the PTBD group. These findings provide important evidence supporting the use of EUS‐BD as a first‐line alternative to PTBD in patients with malignant biliary obstructions. Further prospective, randomized, multicenter studies are needed to confirm these findings and to explore the role of operator expertise and institutional experience in optimizing patient outcomes.

## Author Contributions

**Thomas Roland Heiduk** and **Ahmad Amanzada** conceived and designed the study. Thomas Roland Heiduk and **Ahmad Amanzada** performed the data analysis. **Thomas Roland Heiduk**, **Ahmad Amanzada**, **Andre Sasse**, **Volker Ellenrieder**, **Lukas Hiebel**, **Richard Friedemann Knoop**, and **Golo Petzold** collected the data and co‐wrote the manuscript with critical input from all authors.

## Conflicts of Interest

The authors declare no conflicts of interest.

## Funding

This study was not supported by any sponsor or funder.

## Ethics Statement

The Ethics Committee of the University Medical Center Göttingen waived the requirement for additional written informed consent for this retrospective study. The human study received final approval from the local Ethics Committee of the University Medical Center Göttingen (approval number 25/12/22). All procedures were conducted in accordance with the Declaration of Helsinki (2013) and applicable local legislation.

## Consent

All included participants had previously provided written consent via the standard hospitalization agreement, which includes permission for the use of their data in medical research.

## Clinical Trial Registration

Additional registration in a clinical trial registry was not conducted, as this is not required in Germany. Consequently, no registration number is available.

## Data Availability

All relevant data are contained within the article. The original contributions presented in the study are included in the article; further inquiries can be directed to the corresponding authors.
